# Identification and expression profiling of microRNAs in leaf tissues of *Foeniculum vulgare* Mill. under salinity stress

**DOI:** 10.1080/15592324.2024.2361174

**Published:** 2024-06-02

**Authors:** Luis Alberto Bravo-Vázquez, Mariana García-Ortega, Sara Medina-Feria, Aashish Srivastava, Sujay Paul

**Affiliations:** a School of Engineering and Sciences, Tecnologico de Monterrey, San Pablo, Queretaro, Mexico; b Department of Clinical Science, University of Bergen, Bergen, Norway

**Keywords:** Fennel, microRNAs, *in silico*, salt stress, gene regulation, miRNA targets

## Abstract

*Foeniculum vulgare* Mill. commonly known as fennel, is a globally recognized aromatic medicinal plant and culinary herb with widespread popularity due to its antimicrobial, antioxidant, carminative, and diuretic properties, among others. Although the phenotypic effects of salinity stress have been previously explored in fennel, the molecular mechanisms underlying responses to elevated salinity in this plant remain elusive. MicroRNAs (miRNAs) are tiny, endogenous, and extensively conserved non-coding RNAs (ncRNAs) typically ranging from 20 to 24 nucleotides (nt) in length that play a major role in a myriad of biological functions. In fact, a number of miRNAs have been extensively associated with responses to abiotic stress in plants. Consequently, employing computational methodologies and rigorous filtering criteria, 40 putative miRNAs belonging to 25 different families were characterized from fennel in this study. Subsequently, employing the psRNATarget tool, a total of 67 different candidate target transcripts for the characterized fennel miRNAs were predicted. Additionally, the expression patterns of six selected fennel miRNAs (i.e. fvu-miR156a, fvu-miR162a-3p, fvu-miR166a-3p, fvu-miR167a-5p, fvu-miR171a-3p, and fvu-miR408-3p) were analyzed under salinity stress conditions via qPCR. This article holds notable significance as it identifies not only 40 putative miRNAs in fennel, a non-model plant, but also pioneers the analysis of their expression under salinity stress conditions.

## 1. Introduction

MicroRNAs (miRNAs) are small, endogenous, highly conserved non-coding RNAs (ncRNAs) of approximately 20 to 24 nucleotides (nt) in length.^1^ They exert a pivotal control on the post-transcriptional regulation of gene expression by virtue of their capability to mediate the degradation or translational inhibition of messenger RNAs (mRNAs).^1^ As a matter of fact, plant miRNAs have been widely associated with several biological functions, including development and reproduction,^2^ cell reprogramming,^3^ secondary metabolism,^4^ and biotic and abiotic stress responses.^5^

The initiation of miRNA biogenesis in plants begins at the nucleus with the transcription of miRNA genes (*MIRs*) by the enzyme RNA polymerase II (RNAPII), giving rise to long primary transcripts known as primary-miRNAs (pri-miRNAs). Successive processing involves the cleavage of pri-miRNAs into stem-loop RNA precursors (pre-miRNAs) through the action of DICER-LIKE1 (DCL1), HYPONASTIC LEAVES 1 (HYL1), and SERRATE (SE) enzymes.^6,7^ Following this, DCL1 recognizes and cleaves the hairpin loop of the pre-miRNAs, generating miRNA/miRNA* duplexes (where miRNA refers to the guide strand and miRNA* to the passenger strand). Eventually, the guide strand of the mature miRNA duplex associates with the RNA-Induced Silencing Complex (RISC), forming a miRNA-ribonucleoprotein complex guided by the ARGONAUTE (AGO) protein. This complex effectively interacts with mRNA targets, culminating in the regulatory processes associated with miRNA-mediated gene silencing.^6,7^

The pervasive adoption of small RNA high-throughput sequencing (sRNA-seq) technology has accelerated a notable surge in the identification of miRNAs over the past decade. Concurrently, there has been an apparent escalation in the entries within the miRBase registry, underscoring the expanding repository of documented plant miRNAs.^8^ Besides, the evolutionary conservation of numerous plant miRNAs facilitates the streamlined characterization of miRNA orthologs in novel plant species through the documentation of homologous sequences.^9^ Even so, reliance solely on sequence-based *in silico* homology approaches for the identification of putative miRNAs in new plants may generate false-positive outcomes. Therefore, a comprehensive approach should be adopted, considering not only the primary sequence but also the secondary structures of pre-miRNAs, along with critical parameters such as length, GC content, Minimum Folding Free Energy (MFE), and Minimum Folding Free Energy Index (MFEI). This multifaceted consideration is essential to enhance the precision of computational predictions, enabling the discrimination of miRNAs from other coding RNA or ncRNA entities.^10^ Nevertheless, it is strongly advisable to validate the computationally predicted miRNAs through experimental methods.^11^

Fennel (*Foeniculum vulgare* Mill.), an enduring tropical herb belonging to the Apiaceae (also known as Umbelliferae) family and native to the Mediterranean region, is distinguished by its abundant branches, tender leaves, small yellow flowers, elongated fruits, fragrant scent, and a foliage resembling fine hairs.^12,13^ Moreover, different parts of fennel plant (e.g., seeds, fruits, and leaves) own remarkable antispasmodic, carminative, diuretic, antimicrobial, and antioxidant properties, among others,^14^ due to the presence of several bioactive compounds such as limonene, chlorogenic acid, glycosides of quercetin, rosmarinic acid, estragole, anethole, and kaempferol.^15^

Salinity stress represents a major abiotic factor exerting considerable impact on the growth, development, and productivity of plants, as well as on crop yields. In this context, it has been demonstrated that salinity stress affects biomass, photosynthetic pigments, leaf area, and relative water content in *F. vulgare*.^16,17^ Despite the above, the biological significance of miRNAs in the context of salinity stress in *F. vulgare* remains enigmatic. Therefore, understanding the regulatory mechanisms through which miRNAs operate under salinity stress conditions in this species holds significance for unraveling the molecular intricacies of stress response pathways. Consequently, in this current investigation, the published draft genome sequence of fennel (GenBank assembly accession GCA_003724115.2) facilitated the characterization of numerous miRNAs and their respective targets. Additionally, an analysis of the expression patterns of some of these miRNAs under salinity stress was conducted, aiming to enhance our comprehension of the physiological role played by miRNAs in fennel.

## 2. Materials and methods

### 2.1. Computational prediction of potential fennel miRNAs and their putative pre-miRNAs

**Figure 1.**
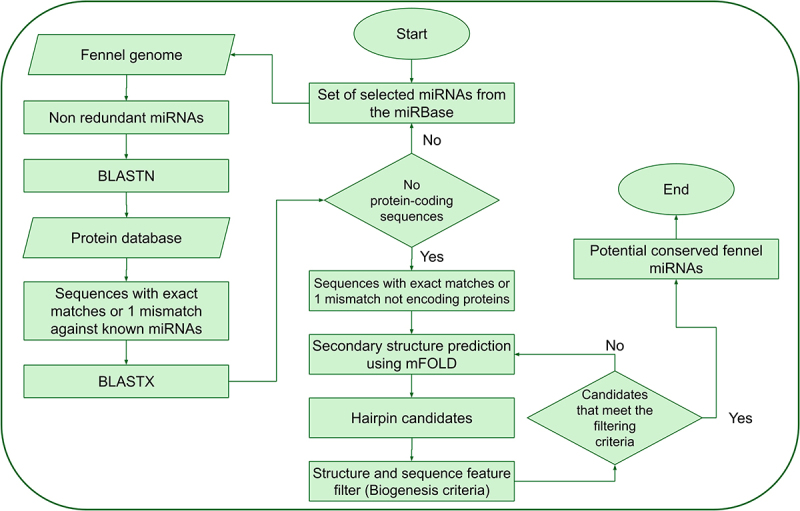
Schematic depiction of the fennel miRNA prediction procedure.

An *in silico* analysis was conducted to predict and identify potential miRNAs in fennel using a reference set of plant miRNAs sourced from the miRBase database.^18^ The reference set comprised a total of 1,216 known mature miRNAs from *Arabidopsis thaliana* (428), *Glycine max* (756), and *Panax ginseng* (32). In brief, the known miRNAs were subjected to BLASTn against the fennel genome, and sequences with exact matches or a maximum of 1 mismatch were manually selected. Potential precursor sequences of approximately 400 nt in length (200 nt upstream and 200 nt downstream of the BLAST hit region) were identified, with the exclusion of protein-coding sequences. The selected precursors’ stable secondary structures were generated using the mFold web server,^19^ and their stability was assessed through established stringent filtering criteria. These criteria included ensuring that (a) the pre-miRNA possessed a stem-loop structure containing the mature miRNA sequence within one arm, (b) the mature miRNA was not presented in the terminal loop of the hairpin structure, (c) the mature miRNA had fewer than nine mismatches with the opposite miRNA* sequence, (d) and the potential stem-loop candidate exhibited a minimum negative MFE (or ΔG, −kcal/mol) coupled with a high MFEI (the MFEI calculation followed the formula provided below). The procedure for the computational prediction of miRNAs in fennel is illustrated in Figure 1.

$$\mathrm{MFEI}=\frac{\left(\mathrm{MFE}/l\mathrm{enght}\;of\;the\;RNA\;s\mathrm{equence}\right)\;x\;100}{\%\mathrm{GC}\;\mathrm{content}}$$

### 2.2. Phylogenetic and conservation analysis of fennel miRNA precursors

For the conservation analysis of the predicted fennel pre-miRNA candidates, the miRNA precursor sequences from various plant species were recovered,^18^ including *Amborella trichopoda* (atr), *Arabidopsis lyrata* (aly), *Arabidopsis thaliana* (ath), *Asparagus officinalis* (aff), *Brachypodium distachyon* (bdi), *Brassica napus* (bna), *Carica papaya* (cpa), *Citrus sinensis* (csi), *Cucumis melo* (cme), *Fragaria vesca* (fve), *Glycine max* (gma), *Linum usitatissimum* (lus), *Malus domestica* (mdm), *Nicotiana tabacum* (nta), *Oryza sativa* (osa), *Theobroma cacao* (tcc), and *Vitis vinifera* (vvi). After that, multiple sequence alignment and phylogenetic tree assembly (grounded in the Tamura-Nei model with 100 boot-strapped replicates) were executed using the Molecular Evolutionary Genetics Analysis (MEGA) software (version 11.0.11).^20^ Besides, the sequence conservation analysis of three representative predicted fennel pre-miRNAs, i.e., pre-miR156a, pre-miR166a, and pre-miR171a, was carried out using the WebLogo tool,^21^ considering their orthologs. Furthermore, to clarify the highly conserved characteristics of miRNAs across diverse species and their potential for cross-species transferability, a syntenic map was created employing the putative fennel pre-miRNAs against the well-annotated *Daucus carota* (carrot) genome (GenBank assembly accession GCF_001625215.1), a closely related species of fennel.^22,23^

### 2.3. Target prediction of fennel miRNAs and their functional annotations

In order to predict the potential target transcripts of the fennel miRNAs, the “Plant Small RNA Target Analysis Server” (psRNATarget)^24^ was utilized. The search for target transcripts was conducted against the database of *D*. *carota* (that belongs to the same family of fennel, i.e., Apiaceae^25,26^) due to the unavailability of the fennel database on the psRNATarget cDNA library. The specified selection parameters included an expectation value of 3, translation inhibition ranges spanning from 9 to 11 nt, a top target count of 10, a penalty for G:U pair of 0.5, and allowance for 1.5 mismatches in the seed region. Afterward, the protein data for the identified sequences was retrieved using UniProt BLAST. Subsequently, the gene ontology (GO) analysis of the putative fennel targets was performed, elucidating the associated biological processes, cellular components, and molecular functions for each GO term employing the QuickGO tool.^27^ Additionally, to discern the co-regulation among potential targets, a biological network was constructed using Minimum Free Energy (MFE) values of miRNA-target interactions and visualized through the Cytoscape 3.10.1 software.^28^ Lastly, KEGG analysis was executed via the KEGG Automatic Annotation Server (KAAS)^29^ to explore the metabolic pathways and networks modulated by the prospective fennel miRNAs, utilizing the Bi-directional Best Hit (BBH) method.

### 2.4. Plant materials, stress treatment, small RNA extraction, and miRNA expression analysis

Fennel seeds were germinated in sterile paper towel at 25°C in darkness and the seedlings were then transferred into two hydroponic systems with automated LED illumination from iDOO (ID-IG301, Eastvale, CA, USA) containing a culture media prepared by mixing distilled water with a registered commercial hydroponic solution (Interagro, Guadalajara, JAL, Mexico) with a pH adjusted to 7.^30^ The plants were allowed to grow under controlled environmental conditions at 25°C and with a 16 h photoperiod (automatically regulated by the hydroponic system) for 6 weeks; the hydroponic solutions were renewed weekly. Following this, the hydroponic solution for one of the systems was formulated with a concentration of 200 mM NaCl to induce salinity stress, whereas the solution for the other system was prepared without NaCl, serving as the control.

Next, leaves from both stressed and control fennel plants were harvested after a short exposure (24 h) and a long exposure (72 h). Small RNA molecules (<200 nt) were extracted from the leaf tissues using the miRNeasy Mini Kit (Qiagen, Hilden, Germany) according to the manufacturer’s guidelines. Subsequently, both the quality and quantity of the RNA samples were assessed using Nanodrop One (Thermo Scientific, Wilmington, NC, USA). Thereafter, 1 µg of RNA from the individual samples was polyadenylated (using a modified oligo dT primer) and reverse transcribed, employing the mRQ Buffer and enzyme of the Mir-X miRNA First-Strand Synthesis kit (Takara, Tokyo, Japan). The qRT-PCR experiment was conducted utilizing the Step One Real-Time PCR System (Applied Biosystems, Carlsbad, CA, USA) and the Mir-X miRNA TB Green qRT-PCR kit (Takara, Tokyo, Japan).

The entire predicted miRNA sequence of each of the analyzed miRNAs served as the forward primer, while the adapter-specific mRQ3’ primer provided with the kit was used as the reverse primer. Each reaction was prepared in a 12.5 µL volume, comprising 1× SYBR Advantage Premix, 1× ROX dye, 0.2 µM of both forward and reverse primers, and 2 µL of the first-strand cDNA. A total of six selected miRNAs (i.e., fvu-miR156a, fvu-miR162a-3p, fvu-miR166a-3p, fvu-miR167a-5p, fvu-miR171a-3p, and fvu-miR408-3p), previously documented to play pivotal roles in both biotic and abiotic stress responses^31^ were chosen for the qRT-PCR experiment. The qPCR conditions comprised an initial denaturation at 95°C for 10 s, 45 cycles of denaturation at 95°C for 5 s, annealing at 63°C for 20 s, and concluding with a dissociation curve consisting of 95°C for 30 s, 55°C for 20 s, and 95°C for 20 s. The relative fold change values were calculated through the application of the comparative Ct method, also known as the delta-delta Ct method (2^−ΔΔCT^). All the qPCR reactions were performed using three biological replicates and two technical replicates.

## 3. Results

### 3.1. Characterization of fennel miRNAs and their candidate precursors

**Figure 2.**
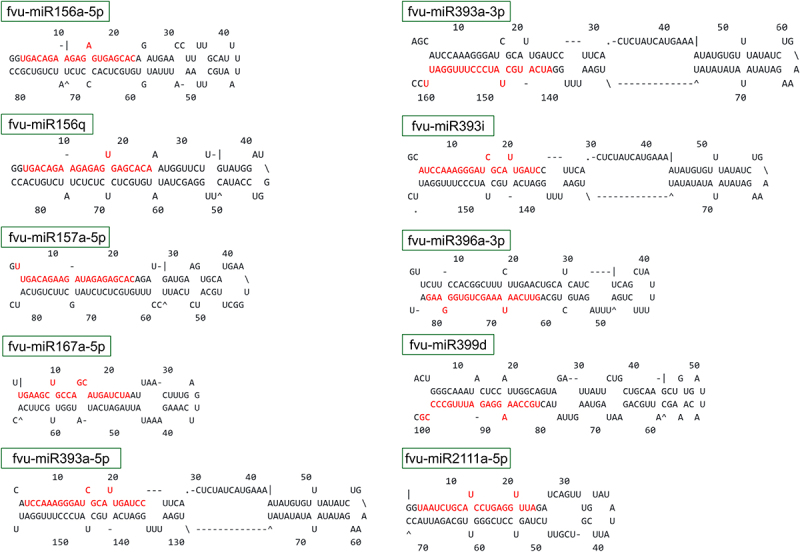
Secondary stem-loop structures of the top ten putative fennel pre-miRNAs. The corresponding mature miRNAs are indicated in red font.

In this investigation, employing computational strategies and sticking to rigorous filtering criteria, a set of 40 potential miRNAs distributed across 25 families was identified (Table 1). The predominant length among the identified fennel miRNAs was 21 nt. The precursors of these fennel miRNAs exhibited substantial variability in size, ranging from 68 to 210 nt, with an average length of 114 ± 38 nt. The fennel miRNA fvu-miR319c displayed the longest precursor with 210 nt, whereas fvu-miR167a-5p exhibited the shortest one with 68 nt. Besides, the MFE values of the precursors ranged from −88.40 to −30.00 kcal/mol, with an average of −47.27 ± 13.87 kcal/mol. Concurrently, the MFEI values varied from 0.71 to 1.47, averaging 1.03 ± 0.19. The predicted secondary structures of fennel miRNA precursors featuring higher MFEI values (top 10) are depicted in Figure 2.

**Table 1.** Candidate miRNAs identified in fennel through bioinformatic methods.

Predicted miRNA	LM* (nt)	Query miRNA	Number of mismatches	miRNA sequence*	miRNA accession	Strand	Location	LP* (nt)	GC %	MFE (ΔG)	MFEI
fvu-miR156a-5p	20	ath-miR156a-5p	0	UGACAGAAGAGAGUGAGCAC	MI0000178	Plus/Plus	5’	82	42.68	−43.80	1.25
fvu-miR156q	21	gma-miR156q	1	UGACAGAAGAGAGUGAGCAC**A**	MI0019748	Plus/Plus	5’	85	45.88	−56.20	1.44
fvu-miR157a-5p	21	ath-miR157a-5p	0	UUGACAGAAGAUAGAGAGCAC	MI0000184	Plus/Plus	5’	85	43.53	−45.70	1.24
fvu-miR159a	21	ath-miR159a	0	UUUGGAUUGAAGGGAGCUCUA	MI0000189	Plus/Minus	3’	170	44.71	−78.70	1.04
fvu-miR160a-5p	21	ath-miR160a-5p	0	UGCCUGGCUCCCUGUAUGCCA	MI0000190	Plus/Minus	5’	87	55.17	−45.50	0.95
fvu-miR162a-3p	21	ath-miR162a-3p	0	UCGAUAAACCUCUGCAUCCAG	MI0000194	Plus/Minus	3’	96	43.75	−38.00	0.90
fvu-miR164a	21	ath-miR164a	0	UGGAGAAGCAGGGCACGUGCA	MI0000197	Plus/Plus	5’	138	48.55	−49.50	0.74
fvu-miR166a-3p	21	ath-miR166a-3p	0	UCGGACCAGGCUUCAUUCCCC	MI0000201	Plus/Plus	3’	88	50.00	−47.40	1.08
fvu-miR167a-5p	21	ath-miR167a-5p	0	UGAAGCUGCCAGCAUGAUCUA	MI0000208	Plus/Plus	5’	68	33.82	−30.10	1.31
fvu-miR169a-3p	20	ath-miR169a-3p	0	GGCAAGUUGUCCUUGGCUAC	MI0000212	Plus/Minus	3’	154	40.91	−50.50	0.80
fvu-miR169b-5p	21	ath-miR169b-5p	0	CAGCCAAGGAUGACUUGCCGG	MI0000976	Plus/Plus	5’	107	42.06	−47.60	1.06
fvu-miR169h	21	ath-miR169h	0	UAGCCAAGGAUGACUUGCCUG	MI0000982	Plus/Plus	5’	91	49.45	−41.90	0.93
fvu-miR171a-3p	21	ath-miR171a-3p	0	UGAUUGAGCCGCGCCAAUAUC	MI0000214	Plus/Minus	3’	82	43.90	−36.10	1.00
fvu-miR171b-3p	21	ath-miR171b-3p	0	UUGAGCCGUGCCAAUAUCACG	MI0000989	Plus/Minus	3’	88	38.64	−33.90	1.00
fvu-miR171k-3p	21	gma-miR171k-3p	0	UUGAGCCGCGCCAAUAUCACU	MI0018667	Plus/Plus	3’	93	43.01	−38.80	0.97
fvu-miR171l	21	gma-miR171l	0	CGAUGUUGGUGAGGUUCAAUC	MI0018687	Plus/Minus	5’	89	46.07	−35.70	0.87
fvu-miR172a	21	ath-miR172a	0	AGAAUCUUGAUGAUGCUGCAU	MI0000215	Plus/Plus	3’	80	35.00	−30.00	1.07
fvu-miR172c	21	ath-miR172c	0	AGAAUCUUGAUGAUGCUGCAG	MI0000991	Plus/Plus	3’	143	44.06	−65.20	1.03
fvu-miR319a	21	ath-miR319a	0	UUGGACUGAAGGGAGCUCCCU	MI0000544	Plus/Plus	3’	179	45.25	−88.40	1.09
fvu-miR319c	20	gma-miR319c	0	UUGGACUGAAGGGAGCUCCU	MI0001789	Plus/Minus	3’	210	45.71	−83.60	0.87
fvu-miR390a-3p	21	ath-miR390a-3p	0	CGCUAUCCAUCCUGAGUUUCA	MI0001000	Plus/Minus	3’	127	43.31	−52.30	0.95
fvu-miR390a-5p	21	ath-miR390a-5p	0	AAGCUCAGGAGGGAUAGCGCC	MI0001000	Plus/Plus	5’	117	39.32	−43.70	0.95
fvu-miR393a-3p	21	ath-miR393a-3p	1	AUCAUGCUAUC**C**CUUUGGAUU	MI0001003	Plus/Minus	3’	163	28.83	−64.50	1.37
fvu-miR393a-5p	22	ath-miR393a-5p	0	UCCAAAGGGAUCGCAUUGAUCC	MI0001003	Plus/Minus	5’	159	27.67	−64.50	1.47
fvu-miR393i	22	gma-miR393i	1	**A**UCCAAAGGGAUCGCAUUGAUC	MI0021710	Plus/Minus	5’	161	28.57	−64.50	1.40
fvu-miR394a	20	ath-miR394a	1	UUGGCAUUCUGU**U**CACCUCC	MI0001005	Plus/Plus	5’	81	45.68	−39.20	1.06
fvu-miR395a	21	ath-miR395a	0	CUGAAGUGUUUGGGGGAACUC	MI0001007	Plus/Plus	3’	116	43.97	−49.70	0.97
fvu-miR396a-3p	21	ath-miR396a-3p	0	GUUCAAUAAAGCUGUGGGAAG	MI0001013	Plus/Plus	3’	84	40.48	−41.10	1.21
fvu-miR396a-5p	21	ath-miR396a-5p	0	UUCCACAGCUUUCUUGAACUG	MI0001013	Plus/Minus	5’	100	39.00	−37.40	0.96
fvu-miR398a-3p	21	ath-miR398a-3p	0	UGUGUUCUCAGGUCACCCCUU	MI0001017	Plus/Minus	3’	87	44.83	−42.00	1.08
fvu-miR398c	21	gma-miR398c	0	UGUGUUCUCAGGUCGCCCCUG	MI0017847	Plus/Minus	3’	85	47.06	−34.10	0.85
fvu-miR399d	21	ath-miR399d	0	UGCCAAAGGAGAUUUGCCCCG	MI0001023	Plus/Plus	3’	102	43.14	−49.20	1.12
fvu-miR399i	21	gma-miR399i	0	UGCCAAAGGAGAAUUGCCCUG	MI0031044	Plus/Minus	3’	75	49.33	−38.10	1.03
fvu-miR408-3p	21	ath-miR408-3p	0	AUGCACUGCCUCUUCCCUGGC	MI0001080	Plus/Plus	3’	90	53.33	−44.90	0.94
fvu-miR828	22	ath-miR828	0	UCUUGCUUAAAUGAGUAUUCCA	MI0005384	Plus/Plus	5’	109	35.78	−37.00	0.95
fvu-miR846	21	ath-miR846	1	UUGAAUUG**U**AGUGCUUGAAUU	MI0005402	Plus/Plus	5’	153	33.33	−39.70	0.78
fvu-miR858a	21	ath-miR858a	1	UUUCGUUGUCUGUUCG**G**CCUU	MI0005435	Plus/Plus	5’	203	30.54	−44.50	0.72
fvu-miR2111a-5p	21	ath-miR2111a-5p	0	UAAUCUGCAUCCUGAGGUUUA	MI0010630	Plus/Minus	5’	73	42.47	−34.00	1.10
fvu-miR2118	22	pgi-miR2118	1	UUUCCUAUUCC**G**CCCAUCCCAU	MI0021292	Plus/Plus	3’	105	34.29	−39.00	1.08
fvu-miR2934-3p	21	ath-miR2934-3p	1	CAUCCAAGGUGU**G**UGUAGAAA	MI0013364	Plus/Plus	3’	153	41.18	−44.60	0.71

* LM: length of the mature miRNAs, * The mismatches in the predicted fennel miRNAs are underlined and appear in bold text, * LP: length of the precursors.

### 3.2. Conservation analysis of fennel miRNAs and their putative precursors

**Figure 3.**
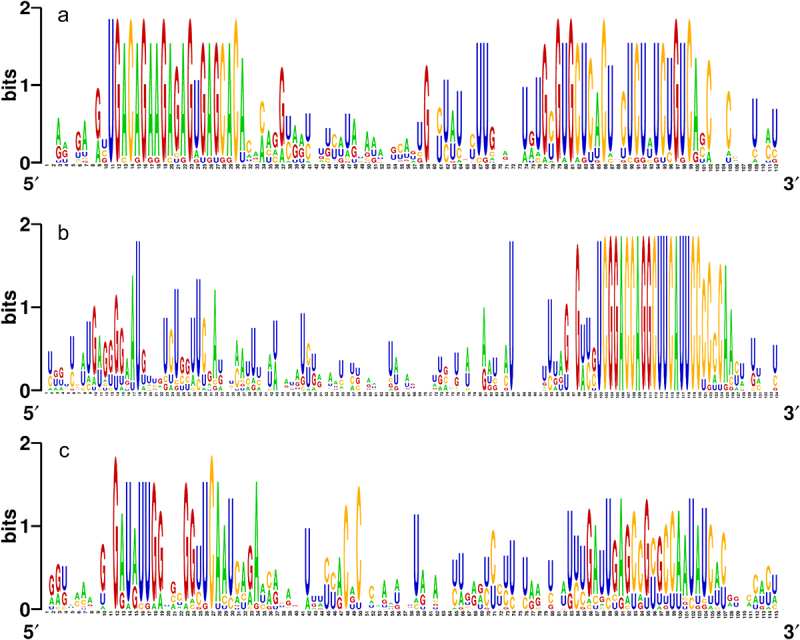
Graphical representation (WebLogo) of the conserved nucleotide sequences among the pre-miRNA orthologs of (a) pre-miR156a, (b) pre-miR166a, and (c) pre-miR171a.

The evolutionary relationships among miRNAs can be explored due to the conserved nature of pre-miRNAs and mature miRNAs. As a matter of fact, search approaches for homologous sequences rely on known miRNAs and pre-miRNAs as references.^32^ As depicted in Figure 3, high conservation of sequence was observed among the orthologs of a set of representative fennel pre-miRNAs comprised of pre-miR156a, pre-miR166a, and pre-miR171a.

**Figure 4.**
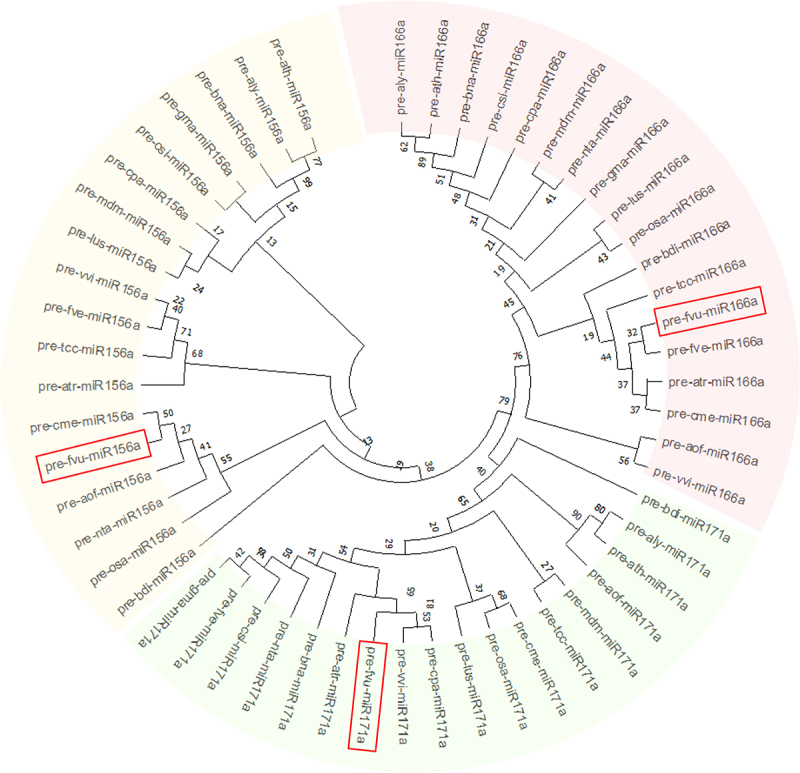
Phylogenetic assessment was conducted on the putative fennel miRNAs fvu-miR156a, fvu-miR166a, and fvu-miR171a (emphasized within the red squares) using their predicted precursor sequences.

**Figure 5.**
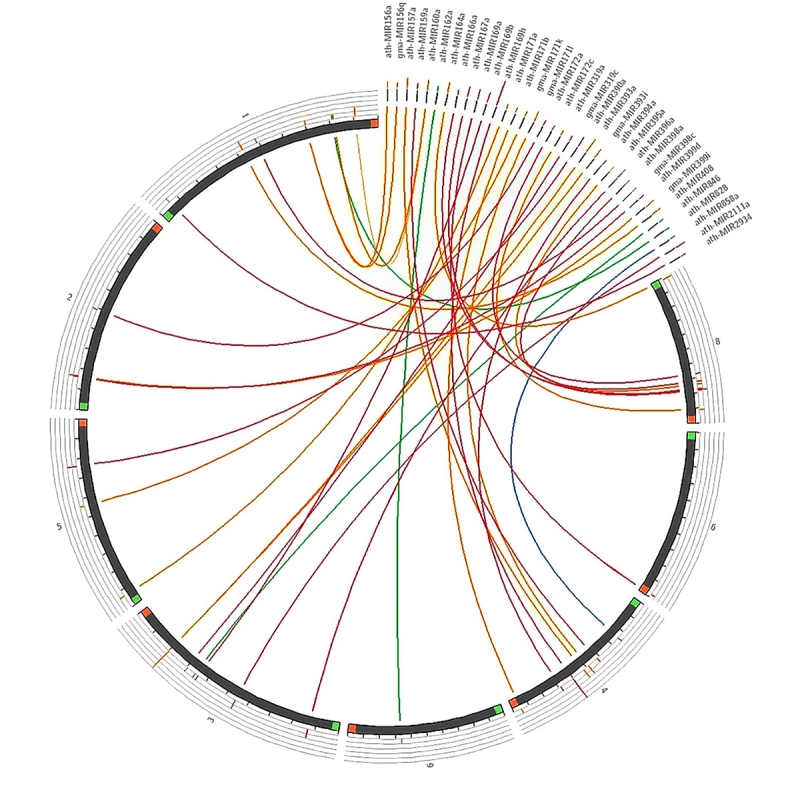
Synteny map comparing candidate fennel miRNAs with the well-annotated genome of a closely related species, carrot (*D. carota*).

Additionally, the phylogenetic analysis of the set of representative pre-miRNAs suggested that fvu-miR156a is closely related to the miRNA orthologs present in melon (cme-miR156a), asparagus (aof-miR156a), tobacco (nta-miR156a), and rice (osa-miR156a); while fvu-pre-miR171a is closer to papaya, common grape vine, and amborella (cpa-miR171a, vvi-miR171a, and atr-miR171a). Additionally, fvu-miR166a showed a close phylogenetic relationship with *Brachypodium distachyon* (bdi-miR166a), cacao (tcc-miR166a), wild strawberry (fve-miR166a), amborella (atr-miR166a), and melon (cme-miR166a) (Figure 4). On the other hand, the comparative synteny map demonstrated the broad distribution of the prospective fennel miRNA orthologs within the carrot genome, illustrating their ability to transfer across species during evolutionary processes (Figure 5).

### 3.3. Predicted targets for fennel miRNAs and their functional annotations

**Figure 6.**
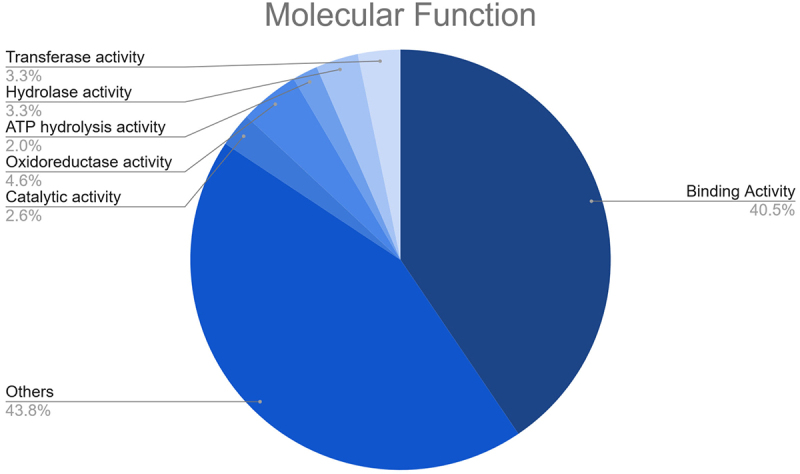
Results of the GO enrichment analysis of the putative fennel miRNA targets related to Molecular Function.

In this study, 67 different potential target transcripts of fennel miRNAs were predicted. Such target transcripts include a number of important molecular entities, such as SBP-type domain-containing proteins, HTH myb-type domain-containing proteins, transcription factor GAMYB-like, auxin response factors, NAC domain-containing proteins, F-box domain-containing proteins, magnesium transporter, alpha-glucosidase, cytochrome P450, ceramidase, among others. Subsequently, a Gene Ontology (GO) analysis of the predicted targets was performed to gain a more profound insight into the plausible functions of the fennel miRNAs. As well, this analysis helped to unravel the regulatory network of the miRNA genes involved in biological mechanisms, molecular functions, and cellular components (Figure 5). The GO enrichment analysis indicated that the fennel miRNA targets are involved in diverse molecular functions, including binding activity (e.g., DNA binding, protein binding, and metal ion binding), transferase activity, hydrolase activity, ATP hydrolysis activity, oxidoreductase activity, and catalytic activity (Figure 6).

**Figure 7.**
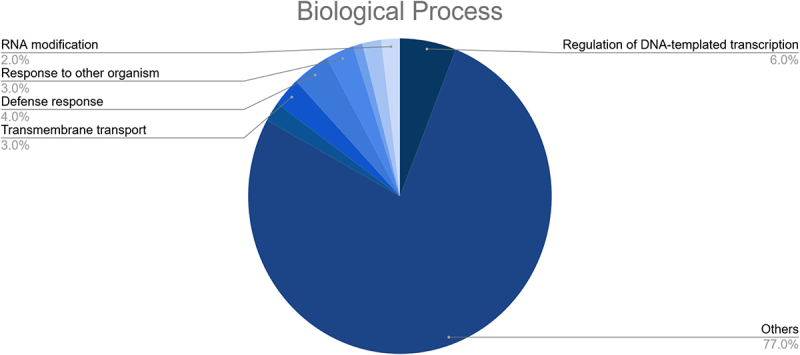
Results of the GO enrichment analysis of the putative fennel miRNA targets related to Biological Process.

**Figure 8.**
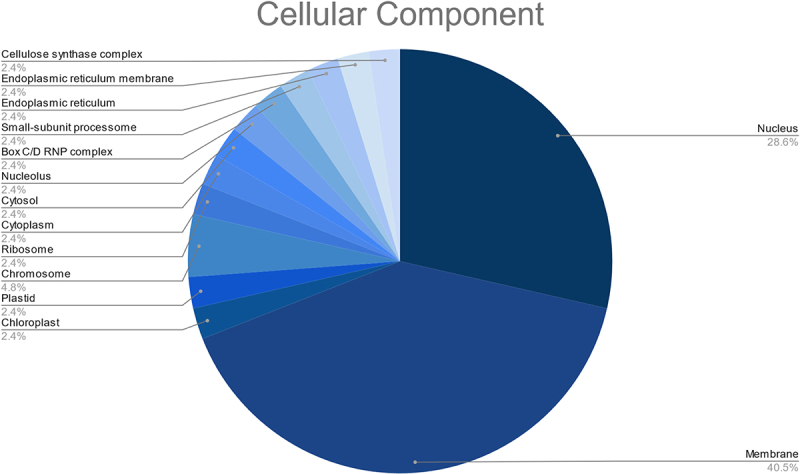
Results of the GO enrichment analysis of the putative fennel miRNA targets related to Cellular Component.

**Figure 9.**
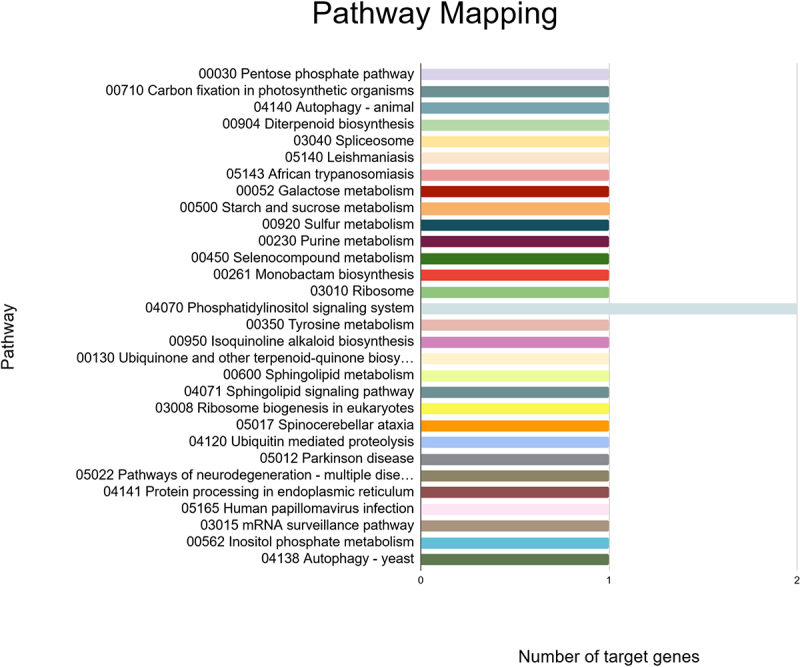
KEGG pathways of the predicted fennel miRNA targets that were mapped using the KAAS tool.

**Figure 10.**
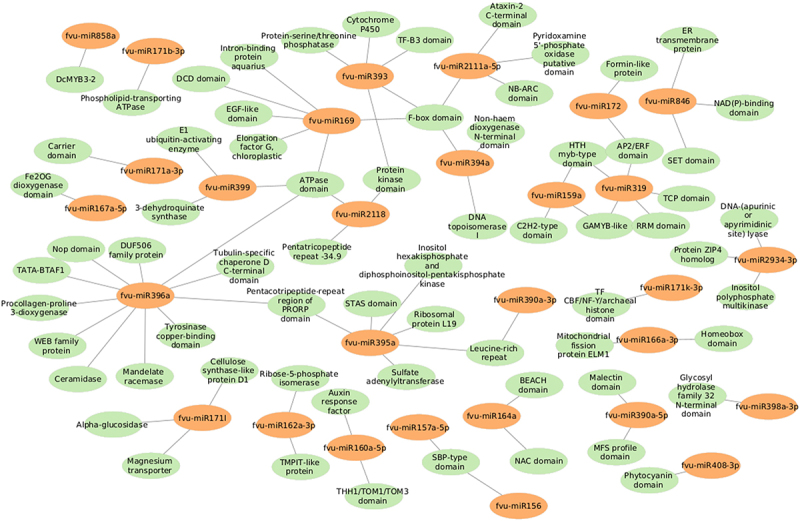
Network interactions based on minimum free energy (MFE) between prospective fennel miRNAs (orange circles) and their corresponding potential targets (green circles).

These targets were also found to participate in a variety of biological processes like regulation of DNA-templated transcription, RNA modification, response to other organism, defense response, and transmembrane transport (Figure 7). Meanwhile, the most enriched cellular components were found to be the membrane, nucleus, cellulose synthase complex, endoplasmic reticulum membrane, endoplasmic reticulum, nucleolus, cytosol, cytoplasm, amongst others (Figure 8). Besides, the KEGG analysis implied that the putative fennel miRNA targets are involved in 30 different metabolic pathways, being the “Phosphatidylinositol signaling system” the most enriched one (Figure 9). Additionally, the gene network analysis illustrated in Figure 10 shows the coordinated regulation of multiple potential fennel miRNA target genes.

### 3.4. Expression profiling of fennel miRNAs under salinity stress

**Figure 11.**
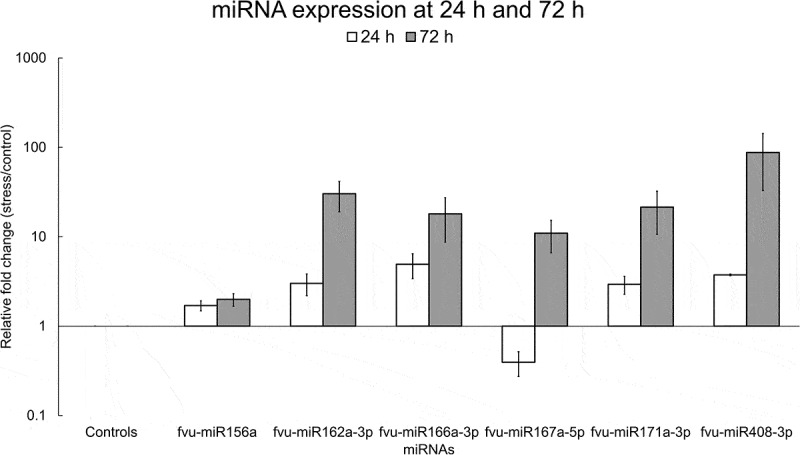
Relative fold change of selected fennel miRNAs under salinity stress at 24 h and 72 h. The fold change was calculated using the delta-delta CT method, U6 snRNA was utilized as the normalization control, and the values were normalized relative to the control condition (set as 1). Error bars indicate the standard error of the biological replicates.

To explore the impact of salinity stress on the expression of selected fennel miRNAs (fvu-miR156a, fvu-miR162a-3p, fvu-miR166a-3p, fvu-miR167a-5p, fvu-miR171a-3p, and fvu-miR408-3p) in leaves, a qRT-PCR experiment was conducted. The results revealed differential expression of all six miRNAs in response to salinity stress both at 24 h and 72 h. The expression levels of fvu-miR156a, fvu-miR162a-3p, fvu-miR166a-3p, fvu-miR171a-3p, and fvu-miR408-3p were upregulated at 24 h, while fvu-miR167a-5p was downregulated when compared to the fennel plants that were not subjected to salinity stress. Later, at 72 h, the expression levels of all the studied miRNAs were found to be upregulated (Figure 11).

## 4. Discussion

The significant functions of miRNAs in plant development, growth, and adaptation to both biotic and abiotic stress have turned them into compelling molecular subjects of research, especially in the pursuit of miRNA-based strategies for plant improvement.^33^ Despite the fact that miRNAs have been extensively investigated in various key crops and model plants, such as *Arabidopsis* ^34^ and soybean,^35^ the functional roles of these master regulators of gene expression remain elusive in non-model plant species like fennel. However, recent advances in the assembly of the fennel genome^26^ and leaf transcriptome^25^ have allowed us to advance in the prediction and functional characterization of the miRNAs belonging to this aromatic medicinal plant. In fact, the putative fennel miRNAs identified in this current investigation, as well as their corresponding precursors, presented high sequence conservation with their orthologs from different monocot and dicot plant species. This finding suggests that miRNAs are universally conserved between monocotyledonous and dicotyledonous species,^36^ implying that they may fulfill similar physiological functions.

The precursors of the fennel miRNAs predicted herein exhibited a wide range of sizes, spanning from 68 to 210 nt and displayed stable stem-loop secondary structures, consistent with findings reported in numerous other recently studied non-model plant species including cranberry,^37^ neem,^38^ and *Sorghum bicolor*.^39^ Remarkably, all the predicted precursor structures of the fennel miRNAs showed high MFEI values that ranged from 0.71 to 1.47, with an average of 1.03, which is greater than the MFEI values associated with other RNA species (e.g., mRNAs: 0.62–0.66, tRNAs: 0.64, or rRNAs: 0.59).^40^ In our assessment, a notable predominance of uracil at the initial position of the predicted miRNAs was also acknowledged. This observation reinforces the reliability of our results and aligns with previous findings emphasizing the pivotal role of uracil at the start of the mature miRNA in governing miRNA-mediated regulatory mechanisms.^41,42^

In line with previous findings, the majority of predicted targets of fennel miRNAs are transcription factors primarily associated with plant growth, developmental patterning, cell differentiation, and stress responses.^43^ Several of the predicted transcripts targeted by the putative fennel miRNAs corresponded to transcription factors SBP/SPL, which have fundamental participation in flowering time, changes from the vegetative phase to the reproductive phase, and juvenile-to-adult transition. In concordance with our results, SBP/SPL transcription factors are reported to be targeted by the miR156 family, mainly by miR156 and miR157.^44^ In addition, MYB transcription factors were predicted as targets of fennel miRNAs. These transcription factors play crucial roles in regulating various functions, including the regulation of gene expression in diverse pathways that include secondary metabolism, plant hormone signaling, as well as developmental and morphological processes.^45^ Indeed, as herein noticed with the fennel miRNAs, members belonging to the miR159 family regulate developmental phase transitions in plants by targeting genes encoding MYB transcription factors.^46^

Another important group of transcription factors that were detected to be targeted by fennel miR160a-5p are comprised of ARFs; these transcripts play a major role in both auxin biosynthesis and auxin signaling pathway, being key participants in processes like leaf, flower and fruit development, as well as diverse stress responses.^47^ Meanwhile, NAC domain-containing proteins were identified as the plausible targets of fvu-miR164a, which largely agrees with the fact that the miR164-NAC module is widely conserved in plant species and has a crucial role in adaptative stress responses.^48^ These findings shed light on the potential biological significance of fennel miRNAs, indicating the conservation of such functions across species and highlighting transcription factors as their primary targets.

Interestingly, plants possess specialized mechanisms to mitigate the effects of salinity stress, with miRNAs playing a pivotal role in their regulation. Mechanistically, salt stress can induce the expression (upregulation) of miRNAs, which in turn downregulate target mRNAs encoding proteins that have negative effects on the plant’s response to high salinity. Contrarywise, the downregulation of other miRNAs leads to the accumulation of their target mRNAs, which positively contribute to stress adaptation.^49^ In this context, our results show that fvu-miR156a and fvu-miR162a-3p were upregulated under salinity conditions both at 24 h and 72 h, while fvu-miR167a-5p was upregulated at 72 h. These observations agree with the outcomes reported in previous articles. For instance, Sun et al.^50^ elucidated that miR156, miR162, and miR167 (among other miRNAs) were upregulated in switchgrass under higher salt concentrations. As well, it has been demonstrated that the transgenic expression of zma-miR156 enhanced salt and osmotic tolerance in tobacco via downregulating NtSPL2 and NtSPL9.^51^ thus indicating that the upregulation of miR156 is highly relevant in some plant species to overcome salinity stress. Likewise, Luo et al.^52^ detected that miR162 was upregulated at 24 h and 48 h in *Poa pratensis* callus subjected to salt stress. Moreover, miR167 was detected to be upregulated under salinity stress conditions in *Tamarix chinensis*, causing the downregulation of ARFs such as TcARF6.^53^

On the other hand, fvu-miR166a-3p was upregulated in fennel plants that were subjected to salinity stress. Although it has been widely reported that diverse members of the miR166 family are downregulated under salinity stress in several plants, including guava^54^ and *Arabidopsis*,^55^ other reports on soybean^56^ and highbush blueberry^57^ imply that some members of the same miRNA family become upregulated as a result of salt stress. Furthermore, as acknowledged with fvu-miR171a-5p, Liu et al.^58^ and Deng et al.^59^ stated that miR171 is upregulated during salinity stress in *Arabidopsis* and barley, respectively. In regard to miR408, Ma et al.^60^ unveiled that this miRNA becomes upregulated under high salinity conditions in *Arabidopsis* and that its overexpression enhances the tolerance to salinity stress. Such observations coincide with the fact that fvu-miR408-3p was also upregulated in fennel after the induction of salinity stress. In fact, the overexpression of miR408 encloses a prospective use in plant improvement since transgenic tobacco plants expressing the *Salvia miltiorrhiza* miR408 displayed enhanced seed germination and decreased accumulation of reactive oxygen species under salt stress conditions.^61^ Overall, the findings discussed in the previous lines shed light on the crucial role of fennel miRNAs in response to salinity stress. However, further exploration is required to define the complex miRNA-mRNA interactions and extensive regulatory networks that occur in this medicinal plant.

## 5. Conclusions

The regulatory roles of miRNAs at the post-transcriptional level significantly influence the overall gene regulatory network in plants, and the study of the interplay between miRNAs and their corresponding mRNA targets in non-model plants could significantly benefit the progress in the understanding of miRNAs’ biological functions. Over the last years, a growing interest in the computational identification of miRNAs has emerged due to the availability of advanced bioinformatic tools and sequence resources in public databases. This study represents one of the first analyses of miRNAs and their targets in fennel, where 40 potential miRNAs from 25 families were identified using rigorous filtering criteria and homology-based approaches. Additionally, 67 candidate target transcripts were identified for the predicted fennel miRNAs, providing insights into the regulatory roles of these master regulators of gene expression. Notably, six of the predicted miRNAs were confirmed through qPCR experiments, validating their existence and expression in fennel under both control and salinity stress conditions. As well, the current study on the effects of salinity stress on the expression levels of the selected fennel miRNAs enhances our understanding of their functional roles under stress conditions. It is worth noting that the application of artificial miRNA technology and CRISPR/Cas-based methods targeting miRNAs for crop improvement have shown promising results, and this communication also paves the way for future research on salinity stress tolerance in fennel by potentially using such technologies. Overall, this report contributes to the understanding of miRNA-mediated regulatory mechanisms in fennel and provides a foundation for future research on crop enhancement via applying miRNA-based strategies.

## 6. Future perspectives

It is worth emphasizing that fennel is a plant that has been relatively underexplored in terms of its genetic makeup,^26^ with only a limited number of studies on its genetic composition and even fewer investigations focused on the non-coding regions of its genome. For instance, Attia et al.^62^ elucidated that, under salinity stress conditions, *spermine synthase 1* (*SPMS1*), *ornithine decarboxylase 1* (*ODC1*), and *arginine decarboxylase* (*ADC1*) were upregulated in fennel hypocotyls and cotyledons, while *S-adenosylmethionine decarboxylase 1* (*SAMDC1*) was downregulated in fennel radicle. Moreover, when fennel seedlings were subjected to a treatment combination of salt and gibberellic acid (GA), *ODC1* was the only gene overexpressed in hypocotyls and cotyledons, whereas *ADC1* was upregulated in hypocotyls, cotyledons, and radicle.^62^ Although the aforementioned study did not delve into the molecular mechanisms underlying the observed changes in gene expression, the widely reported regulatory roles of miRNAs in salt stress responses and phytohormone signaling suggest the necessity of investigating the molecular association of miRNAs with stress responses against high salinity in fennel, as well as the interplay between fennel miRNAs, GA perception, and signal transduction pathway.

As previously mentioned, fennel represents a highly abundant source of health-promoting bioactive compounds. Consequently, comprehending the elements that regulate secondary metabolism pathways at the transcriptome level could be of utmost importance for effectively manipulating the genetic makeup of fennel and enhancing the production of phytochemicals endowed with therapeutic properties. In this regard, at least three fennel genes have been reported to participate in the biosynthesis of t-anethole, one of the most representative components of fennel essential oils with antimicrobial and anticancer activities.^63–65^ These genes are associated with transcripts encoding the enzymes t-anol/isoeugenol synthase (IGS1), t-anol/isoeugenol O-methyltransferase (AIMT1), and phenylalanine ammonia-lyase (PAL).^25,66^ Particularly, Abdollahi et al.^66^ reported that PAL and IGS1 exhibited significant upregulation 24 hours post-treatment with methyl jasmonate, coinciding with an elevation in the production of t-anethole. Consistently, these findings denote that conducting a thorough investigation into the miRNA-mediated regulation of transcripts encoding the IGS1, AIMT1, and PAL enzymes could help to develop miRNA-based methods to enhance the production of t-anethole in fennel. Remarkably, such an approach could also be extended to explore and improve other metabolic pathways existing within this plant.

Undoubtedly, the absence of a well-annotated genome for fennel poses challenges when predicting miRNA targets and, in this current research, the genome of carrot, a plant phylogenetically related to fennel, was used as a reference to predict miRNA targets in fennel. To overcome the limitation of a thoroughly annotated genome from fennel in forthcoming assays, various techniques can be employed to validate the presence of the putative fennel transcripts and proteins predicted herein. For instance, RNA-seq data^67^ specifically obtained from fennel tissues can confirm the expression of the forecasted miRNA targets. Similarly, techniques like qRT-PCR^68^ can help to validate the expression levels of the predicted target genes. As well, mass spectrometry-based proteomics and other quantitative proteomic approaches^69^ might be useful to detect and quantify the presence of target proteins in fennel tissues. In summary, these combined approaches could provide more reliable evidence regarding the interaction between miRNAs and their targets in fennel, even without a fully annotated genome.

Furthermore, it is crucial to acknowledge that a significantly underexplored aspect of fennel pertains to the transgenic approach for the investigation and enhancement of biological processes, encompassing miRNAs and other transcripts. Hence, the utilization of artificial miRNAs and the tailored engineering of *MIRs* through genome editing tools (e.g., CRISPR/Cas) hold great promise for deeper insights into the functions of fennel miRNAs and for generating plants with desired traits, including stress tolerance/resistance. In light of the information discussed throughout the previous sections, we strongly believe that the insights shared in this report will be relevant in laying the groundwork for the development of new research focused on analyzing both the coding and non-coding transcriptome of fennel.

## Supplementary Material

S1_Data of predicted fennel miRNA targets.xlsx
